# Tuning the Electrochemical Properties of Novel Asymmetric Integral Sulfonated Polysulfone Cation Exchange Membranes

**DOI:** 10.3390/molecules26020265

**Published:** 2021-01-07

**Authors:** Ahmet Halil Avci, Cédric Van Goethem, Timon Rijnaarts, Sergio Santoro, Marco Aquino, Gianluca Di Profio, Ivo F. J. Vankelecom, Wiebe M. De Vos, Enrica Fontananova, Efrem Curcio

**Affiliations:** 1 Department of Environmental Engineering, University of Calabria, Via Pietro Bucci CUBO 45A, 87036 Rende, CS, Italy; ahmethalilavci@hotmail.com (A.H.A.); marco.aquino@unical.it (M.A.); efrem.curcio@unical.it (E.C.); 2 Membrane Technology Group (MTG), Division cMACS, Faculty of Bioscience Engineering, KU Leuven, Celestijnenlaan 200F, P.O. Box 2454, 3001 Leuven, Belgium; cedric.vangoethem@kuleuven.be (C.V.G.); ivo.vankelecom@kuleuven.be (I.F.J.V.); 3 Membrane Science & Technology, University of Twente, P.O. Box 217, 7500 AE Enschede, The Netherlands; t.rijnaarts@gmail.com (T.R.); w.m.devos@utwente.nl (W.M.D.V.); 4 Institute on Membrane Technology, National Research Council (ITM-CNR), Via P. Bucci CUBO 17C, 87036 Rende, CS, Italy; g.diprofio@itm.cnr.it

**Keywords:** cation exchange membrane, chlorosulfonation, sulfonated polysulfone, electrochemical characterization, reverse electrodialysis

## Abstract

In this study, novel asymmetric integral cation exchange membranes were prepared by the wet phase inversion of sulfonated polysulfone (SPSf) solutions. SPSf with different degrees of sulfonation (DS) was synthesized by variation in the amount of chlorosulfonic acid utilized as a sulfonating agent. The characterization of SPSf samples was performed using FTIR and ^1^H-NMR techniques. SPSf with a DS of 0.31 (0.67 meq/g corresponding ion exchange capacity) was chosen to prepare the membranes, as polymers with a higher DS resulted in poor mechanical properties and excessive swelling in water. By a systematic study, the opportunity to tune the properties of SPSf membranes by acting on the composition of the polymeric solution was demonstrated. The effect of two different phase inversion parameters, solvent type and co-solvent ratio, were investigated by morphological and electrochemical characterization. The best properties (permselectivity of 0.86 and electrical resistance of 6.3 Ω∙cm^2^) were obtained for the membrane prepared with 2-propanol (IPA):1-Methyl-2-pyrrolidinone (NMP) in a 20:80 ratio. This membrane was further characterized in different solution concentrations to estimate its performance in a Reverse Electrodialysis (RED) operation. Although the estimated generated power was less than that of the commercial CMX (Neosepta) membrane, used as a benchmark, the tailor-made membrane can be considered as a cost-effective alternative, as one of the main limitations to the commercialization of RED is the high membrane price.

## 1. Introduction

Ion exchange membranes (IEMs) have received increasing attention in well-established electromembrane processes (e.g., dialysis [1], electrodialysis [2], and electrolysis [3]) as well as emerging applications (e.g., fuel cells [4], reverse electrodialysis [5], capacitive deionization [6], etc.).

Having fixed charged moieties in their polymeric matrix, IEMs are able to repel ions with the same sign (co-ions) in an electrolytic environment, while oppositely charged ions (counter-ions) are attracted. The exclusion property against a specific charge is referred to as permselectivity, considered as one of the two main performance indicators together with ionic resistance. However, the overall IEM assessment requires the investigation of additional chemical and physical properties. Swelling degree (SD), a parameter strongly affecting the mechanical stability of IEM, increases with increasing ion exchange capacity (IEC), since the elevated hydrophilic fixed charge content of a membrane drastically enhances the interactions of the polymeric chains with water molecules. On the other hand, resistance and permselectivity decrease with a higher IEC because water channels form across the membrane, favoring non-selective ion transport. Moreover, low membrane cost is decisive for affordable technological solutions.

In electromembrane processes, the preparation of an IEM for specific application and operation conditions is vital [7]. For example, in order to boost the power generation of a Reverse Electrodialysis (RED) stack, the mechanical properties of IEMs can be moderated by reducing the cross-linking degree in return for a reduction in ionic areal resistance since RED is operated under relatively mild conditions [8]. On the other hand, for fuel cells, diffusion dialysis, and ion exchange membrane bioreactors, the critical design criteria is permselectivity between counter-ions of different valance [9].

Depending on their structure, the majority of IEMs can be categorized into two coarse group as homogeneous and heterogeneous [10]. In a homogeneous IEM, ion exchange groups chemically bond to the polymeric backbone, while in a heterogeneous IEM these groups are present locally as ion exchange resin particles distributed in an inert binder. Consequently, a heterogeneous IEM with the same ion exchange capacity of a homogeneous IEM results in distinct poor electrochemical properties, especially areal resistance, due to discontinuous and remote ion exchange moieties [11]. In addition, being relatively thick as a natural consequence of housing coarse resin particles increases the resistance of heterogeneous IEMs. However, heterogeneous IEMs have several prominent advantages from a mechanical strength and cost-effectiveness point of view [12]. An order of magnitude reduction in the suitable homogeneous IEM price is required for an economically feasible RED operation [13,14].

Different preparation methods were described in the literature for homogenous IEMs: (i) the polycondensation of a charged monomer, (ii) the functionalization of a prepared polymer film, and (iii) the preparation of membrane from a dissolved polyelectrolyte [15]. Although it is possible to obtain various types of morphologies with the aforementioned preparation techniques, IEMs are recognized as dense charged membranes supported with a woven cloth or a net [7]. In a review covering the properties of major commercial membranes by Xu et al. (2005), the thickness and IEC of homogeneous IEMs ranged between 50 and 200 µm and 0.8 and 2.5 meq·g^−1^, respectively, while the areal resistance varied between 1 and 5 Ω·cm^2^ (with at least 0.85 permselectivity [16]), regarding the electrochemical properties [17].

Phase inversion has been widely adopted for the preparation of asymmetric integral membranes made of a thin selective layer supported on a porous structure. This morphology has opened the doors to a wide range of applications of membrane technology thanks to a balance of the permselective without affecting the mechanical stability of the membrane. To date, the development of IEMS by phase inversion for the RED process is still unexplored.

Based on their functions, ion exchange membranes are classified as (i) cation exchange membranes (CEMs), (ii) anion exchange membranes (AEMs), (iii) amphoteric ion exchange membranes, iv) bipolar ion exchange membranes, and v) mosaic ion exchange membranes [15]. Being a key component of various electromembrane processes, CEMs contain negative fixed charged moieties on their backbone to be able to exclude co-ions present in the facing electrolyte solution. These fixed charges can be -SO_3_^−^, -COO^−^, -PO_3_^2−^, -PHO_2_^−^, -AsO_3_^2−^, -SeO_3_^−^ [15]. Sulfonation is one of the most accepted functionalization because sulfonate groups are easy to introduce into aromatic groups. Moreover, they dissociate easier than carboxylic acid moieties, and the formation of anhydrides on dehydration is more difficult and slower than that of phosphonic acids [18]. The hydrophobic nature of polysulfone (PSf) makes it an attractive candidate as a CEM material. In addition, it is accepted as an engineering plastic for being cheap, commercially available, and well established [10]. Regarding the desired end-product, various sulfonation agents are reported to obtain sulfonated polysulfone (SPSf). Chlorosulfonic acid (CSA) has been classified as an inexpensive, strong sulfonation agent for PSf, while sulfur trioxide/triethylphosphate and trimethylsilyl chlorosulfonate (TMSCS) are relatively expensive and less reactive [19,20]. Although previous researchers have noted ease of the chlorosulfonation in a chlorinated solvent, they also mentioned chain cleavage and a non−homogenous reaction medium as the drawbacks of the reaction [19,20,21,22,23,24].

In this work, novel asymmetric sulfonated polysulfone cation exchange membranes were prepared by taking the requirements of reverse electrodialysis into account. Tailored membranes are expected to fulfill different insufficiencies of the present CEMs. The high cost of ion exchange membranes was addressed by using an economic sulfonation method of an engineering polymer, while the electrochemical properties were optimized by engineering the morphology. It was possible to obtain an asymmetric CEM that fulfils the electrochemical necessity of the RED process considering the permselectivity (0.89–0.99) and resistance (1.7–11.3 Ω∙cm^2^) of previously tested CEMs [16]. In addition, CEMs were prepared by phase inversion, which is a well-established method for the majority of the membrane manufacturing but ion exchange membranes. This approach brings in a massive flexibility to prepare custom-made membranes for special applications such as reverse electrodialysis.

In this study, integral sulfonated PSf cation exchange membranes were developed via phase inversion. The chlorosulfonation of PSf was carried out by changing the CSA/SPSf ratio in the reaction medium. The asymmetric membrane formation abilities of the obtained SPSf with different DSs were studied and the most convenient polymer was chosen to investigate the various wet−phase inversion parameters, such as solvent and co−solvent. Finally, electrochemical characterization in different solutions was carried out for the best performing membrane, with a direct comparison to a commercial membrane.

## 2. Experimental Procedure

### 2.1. Materials

Polysulfone (PSf Udel^®^ P-1700), provided by Solvay, was dried at 100^°^C overnight before use. Chlorosulfonic acid (CSA, 99%) and deuterated dimethyl sulfoxide (DMSO-*d*_6_, 99.9%) were purchased from Sigma-Aldrich. Dichloromethane (DCM, HPLC grade), 1-Methyl-2-pyrrolidinone (NMP, 99%), dimethylformamide (DMF, extra pure), and 2-propanol (IPA, 99.5%) were supplied by Acros Organics. Neosepta CMX cation exchange membranes were obtained from ASTOM Corp. (Tokyo, Japan).

### 2.2. Sulfonation

DCM was used as a sulfonation reaction medium. First, 10 g of PSf was dissolved in 90 mL of DCM and the solution cooled down to 0 °C in an ice bath. CSA was diluted to 10 *w*/*v*% in DCM and then added dropwise into the polymer solution over 30 min. The CSA amount was changed from 1.58 to 2.35 g, corresponding to theoretical maximum degrees of sulfonation ranging from 0.53 to 0.84, respectively. Following the addition of CSA solution, the reaction medium was allowed to warm-up to room temperature and the reaction continued for 3 h more. At the end of the reaction, SPSf—in the form of a sparingly soluble brownish slurry polymer—was spontaneously precipitated in DCM. Then, excess DCM was decanted and 90 mL of DMF was introduced to obtain a clear polymer solution again. Following this, the polymer was precipitated into 500 mL of technical ethanol. The obtained polymer was rinsed with excess demineralized water and then dried at 70 °C under a vacuum overnight.

The chemical structures of the dry powder PSf and SPSf were characterized by Bruker Alpha II FTIR spectroscopy (Billerica, MA, USA). Characteristic peaks belonging to the sulfonate group were studied qualitatively in a wave frequency interval of 4000 to 400 cm^−1^.

SPSf samples with different degrees of sulfonation were prepared for NMR analysis by dissolving the samples in DMSO-*d*_6_. After complete dissolution, the samples were analyzed by Bruker Avance 400 spectrometer protons. A quantitative calculation known as Kopf’s formula was applied to obtain the sulfonation degree [25,26].

### 2.3. Membrane Preparation

Asymmetric integral membranes were prepared using the wet phase inversion method. Two main solvents, DMF and NMP, and one co-solvent, IPA (also acting as a non-solvent), were used in the dope solution. Both the NMP/IPA and DMF/IPA solvent pairs were varied in a solvent/co-solvent ratio from 100/0 *w*/*w* to 70/30 *w*/*w*. Asymmetric membrane films were cast on a glass plate by a doctor blade with a 250 µm initial thickness. Following casting, the glass plates were immersed into a coagulation bath containing technical IPA and 10 min later were transferred and kept in distilled water until further use.

### 2.4. Membrane Characterization

**Figure 1:**
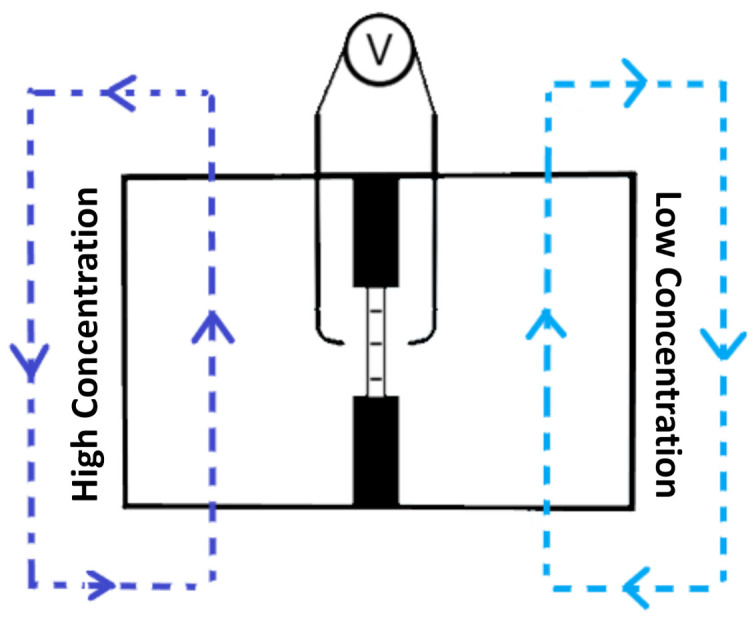
Scheme of two-compartment cell setup for permselectivity characterization.

The membrane potential was determined in a two-compartment cell under salinity gradients of 0.1 M/0.5 M, 0.1 M/1.0 M, or 0.5 M/1.0 M aqueous NaCl solutions (Figure 1). Prior to measurement, the solutions were thermostated to 25 °C and the membranes were conditioned overnight in the low-concentration test solution. In a characterization test, after placing the membrane within the cell NaCl solutions were recirculated through the compartments at a flow rate of 900 mL·min^−1^ by Masterflex peristaltic pumps (Cole-Parmer Srl, Milan, Italy). The membrane permselectivity (α) was calculated from the ratio between the measured membrane potential to the theoretical membrane potential (which represents 100% permselectivity). Electrical potential differences were recorded using a Metrohm Autolab PGSTAT302N() as hardware and NOVA 2.0 as software.

(1) $$\alpha \left(\%\right)=\frac{\Delta V_{measured}}{\Delta V_{theoretical}}$$

The theoretical membrane potential can be calculated by the Nernst equation:

(2) $$\Delta V_{theoretical}=\frac{RT}{zF}ln\left(\frac{C_{2}\gamma_{2}}{C_{1}\gamma_{1}}\right)$$

where *R* is the gas constant (J∙mol^−1^·K^−1^), *T* is the temperature (K), *z* is the valence (−), *F* is the Faraday constant (96,485 s∙A∙mol^−1^), *C* is the concentration (mol∙L^−1^), *γ* is the activity coefficient, and subscripts “1” and “2” indicate the low- and high-concentration solution, respectively.

Table 1 reports the theoretical membrane potentials for the different solution pairs employed in this study.

**Figure 2:**
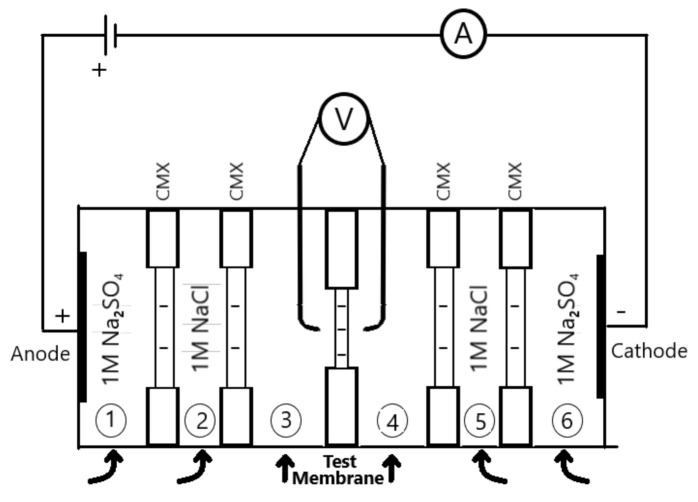
Scheme of the six-compartment resistance characterization cell.

The electrical resistance of the SPSf membranes was characterized in various NaCl solutions by using a six compartment cell as described elsewhere [16]. Figure 2 illustrates the resistance measurement setup supported with two calomel electrodes in the compartment 3 and 4 and two working electrodes in the compartments 1 and 6. Prior to measurement, the membranes were conditioned overnight in the test solution. The membrane under investigation was fitted between compartment 3 and 4, while four CMX membranes were utilized to separate the other compartments. CMX membranes having 99% permselectivity for 0.1 M/0.5 M NaCl solution were preferred to reduce the possible co-ion leakage from neighboring compartments. A total of 1.0 M of Na_2_SO_4_ electrolyte solution was fed into compartments 1 and 6 to avoid toxic chemical production—i.e., Cl_2_ in the use of NaCl—on the electrode surface. In compartments 2 and 5, a shielding solution was fed at the same concentration of the electrolyte solution in compartments 3 and 4 (i.e., 0.1 M NaCl solution in the use of 0.1 M test solution). A Masterflex peristaltic pump was operated to feed the electrolyte, shielding, and test solutions-all thermostated at 25 °C-at a 270 mL·min^−1^ flowrate.

The membrane areal resistance was characterized at three different concentrations: 0.1, 0.5, and 1.0 M NaCl.

DC current ranging between 0 and 15 mA was applied through the working electrodes and the corresponding potential difference in the calomel reference electrodes was recorded. DC resistance (R_DC_) was computed from the slope of a current versus potential difference plot (V = I·R). This resistance comprises membrane resistance (R_M_), boundary layer resistance (R_BL_), and solution resistance (R_S_). To eliminate R_S_, a DC measurement was repeated without the membrane at the same conditions, then R_S_ was subtracted from R_DC_.

The use of alternating current mode allows us to distinguish the membrane resistance and boundary layer resistance, given that the effect of the boundary layer diminishes significantly at high frequencies. Characterization tests were carried out in a frequency range from 10^5^ to 1 Hz. The ohmic resistance of the solution-membrane system was noted where the impedance response intersected the real axis of the Nyquist plot (y = 0). To eliminate R_S_, a blank measurement at the same condition was carried out, then R_S_ was subtracted from the measured AC resistance (R_AC_).

The cross sections of the membranes were observed by a scanning electron microscope JEOL JSM-6010LV (Tokyo, Japan) at a 1000× magnification.

## 3. Results and Discussion

**Figure 3:**
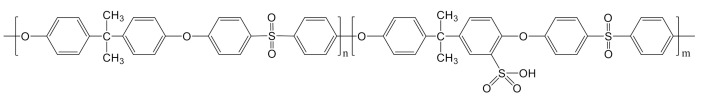
Molecular structure of sulfonated polysulfone (SPSf).

The electrophilic substitution of a sulfonic group on PSf can be achieved up to DS = 2 depending on the reagent ratio present in the reaction medium. As illustrated in Figure 3, substitution mostly occurs on the phenyl ether instead of phenyl sulfone due to the electron-withdrawing character of the SO_2_ group [20].

**Figure 4:**
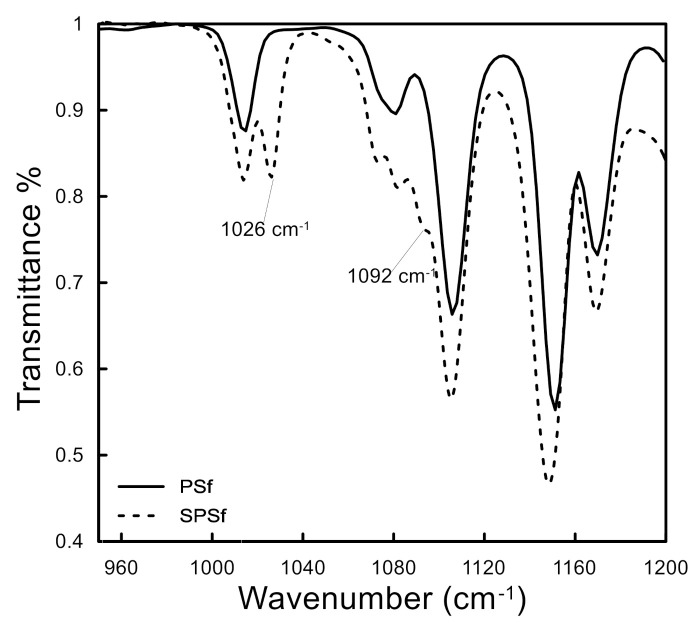
FTIR pattern of PSf and SPSf.

The sulfonation of polysulfone can be confirmed qualitatively by the FTIR spectra. Characteristic peaks of chlorosulfonated PSf are expected to appear near 1027–1030 cm^−1^ and 1070–1096 cm^−1^ related to the symmetric and asymmetric O=S=O stretching of the sulfonate group, respectively [19,28,29]. Figure 4 shows the FTIR patterns of PSf and SPSf in the range for SPSf footprint-i.e., from 950 to 1200 cm^−1^. Characteristic symmetric and asymmetric stretching sulfonate peaks were obtained at 1026 and 1092 cm^−1^, in accordance with the literature [19,28,29].

**Figure 5:**
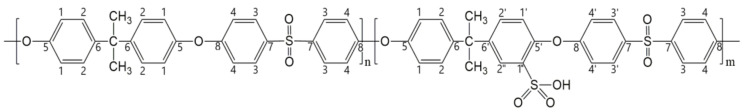
SPSf with numbered protons.

The degree of sulfonation (DS) of synthesized SPSf was quantified by applying Kopf’s formula to the obtained ^1^H-NMR pattern. In the sulfonated polymer units, protons close to the sulfone group split while non-sulfonated units remain unchanged, as illustrated in Figure 5. Area (*A*) under related peaks allows the calculation of the DS with the help of Kopf’s formula:

(3) $$DS=\frac{4\cdot A\left(2^{\prime \prime }\right)}{A\left(3,3\prime \right)}$$

**Figure 6:**
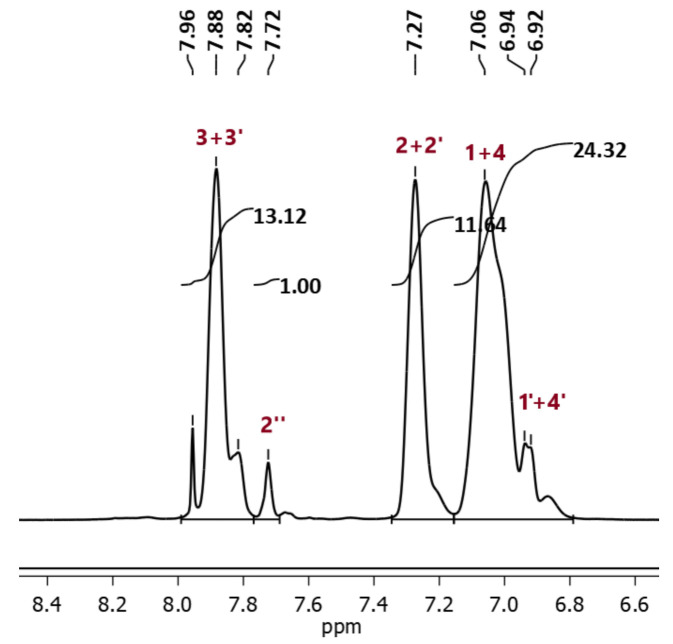
^1^ H-NMR pattern of SPSf-8.

Figure 6 illustrates the obtained ^1^H-NMR pattern of SPSf. Since proton 2′ is a well-resolved singlet peak, it is considered as a reference for the normalization of the other peak areas.

**Figure 7:**
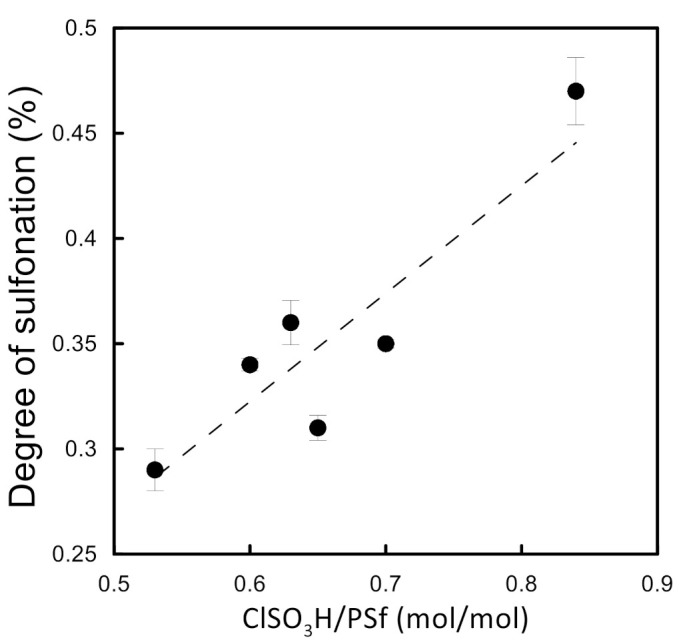
Relation between the reagent ratios and sulfonation degree.

The CSA amount varied from 1.58 g to 2.35 g for 10 g PSf, corresponding to the theoretical maximum degree of sulfonation from 0.53 to 0.84, respectively. Figure 7 illustrates that the effective DS, experimentally determined, ranged between 0.28 and 0.47, indicating that the reaction followed a linear trend for the given interval.

Table 2 compares the Hansen Solubility Parameters of PSf, the sulfonated polymer, and DCM, the solvent where the sulfonation reaction took place. The DS significantly affects the extent of HSP related to polarity (δ_p_) and hydrogen bonding (δ_h_), while the contribution of dispersive forces (δ_d_) remains substantially unchanged.

The affinity between the polymer (*P*) and solvent (*S*) is quantitatively related to the difference ∆*δ_P−S_* between the corresponding Hansen Solubility Parameters, where:

(4) $$\Delta \delta_{P-S}=\sqrt{{\left(\delta_{d,P}-\delta_{d,S}\right)}^{2}+{\left(\delta_{p,P}-\delta_{p,S}\right)}^{2}{\left(\delta_{h,P}-\delta_{h,S}\right)}^{2}}.$$

From the values in Table 2, at higher DS the term ∆*δ_P−S_* increases (for DS = 0, ∆*δ_P−S_* = 2.2 MPa^1/2^; for DS = 0.30, ∆*δ_P−S_* = 2.7 MPa^1/2^; for DS = 1.0, ∆*δ_P−S_* = 4.9 MPa^1/2^) and, therefore, the solvent–polymer affinity decreases. This situation was coherent with empirical observations made during the reaction: half an hour after the CSA addition, the homogeneous reaction medium turned into a cloudy medium from where a brownish SPSf slurry precipitated. Moreover, possible chain cleavage was reported by Baradie et al. (1998) in using strong acid CSA [30].

Due to the precipitation and hydrolysis of the polymer, the sulfonation reaction rate decreases significantly at the solid precipitate/aqueous CSA interface, thus leading to a low DS.

Interestingly, only sulfonated polymers with a relatively low DS (<0.31) were found to be mechanically stable enough for the preparation of self-standing asymmetric integral membranes by wet phase inversion, in line with the objective of this work.

The IEMs used in RED have to maintain their chemical stability for a period of at least 5 years [14]. Considering RED feed solutions, IEMs are exposed to mild natural solutions with a neutral pH and without free chlorine. In addition, there is no pressure difference across the IEMs while the feed temperature is low. Several studies have shown that the sulfonation of polysulfone increases the glass transition temperature while decreasing the decomposition temperature. Lufrano et al. (2000) measured the decomposition temperatures of PSf and SPSf (IEC = 1.19 meq/g) at around 450 °C and 200 °C, respectively [33]. In our study, SPSf has 0.67 meq/g of IEC; in other words, only 31% of the total PSf chains are sulfonated. Therefore, considering the mild conditions of RED and the moderate sulfonation of PSf, the SPSf membranes in this study would expect to maintain their long-term stability.

An attempt to prepare wet phase inversion membranes was made for all the SPSf samples presented in Figure 7. When water was used as a non-solvent, a gel-like polymer precipitate was obtained and self-standing film formation was not possible for any DS. As a result, water was replaced by IPA.

Using SPSf with a DS < 0.31, phase inversion resulted in the formation of white-colored thin membranes after approximately 2 min since the immersion in IPA. After waiting for 10 min, the membranes were immersed in demineralized water. On the other hand, the membranes prepared from SPSf with DS > 0.31 turned into transparent films (indirectly indicating a shift from a porous to a dense membrane morphology) and lost their mechanical stability. On this basis, SPSf with 0.31 DS was selected as the most convenient polymer to prepare membranes by phase inversion; additional parameters—i.e., co-solvent ratio and solvent type and composition—were investigated, as discussed below.

In the phase inversion process, a slow exchange rate between non solvent and solvent typically results in the formation of a dense membrane. Conversely, this study aims at synthetizing membranes with a thin dense top layer (the selective layer) and a sponge-like sublayer structure able to guarantee a high level of permselectivity and a low electrical resistance of the polymeric matrix. An asymmetric integral morphology can be obtained by increasing the demixing rate; this objective can be achieved by adding non-solvent to the solvent mixture to increase its affinity.

In order to carry out a parametric study, membranes were prepared for solvent (DMF or NMP)/co-solvent (IPA) ratios ranging from 100/0 to 70/30. For the solvent/co-solvent binary mixture, the Hansen Solubility Parameters are computed according to the following equation [34,35] and reported in Table 3:

(5) $$\delta_{i,s}=\frac{X_{1}V_{1}\delta_{i,1}+X_{2}V_{2}\delta_{i,2}}{X_{1}V_{1}+X_{2}V_{2}}\;(I\;=\;d,p,h)$$

where the subscripts 1 and 2 mean the solvent the co-solvent, respectively.

The influence of IPA on the thermodynamics of phase demixing can be characterized in terms of Hansen Solubility Parameters difference (∆*δ_NS−SM_*) between non-solvent (*NS*), i.e., IPA and solvent mixture (*SM*), i.e., DMF + IPA or NMP + IPA, defined as:

(6) $$\Delta \delta_{NS-SM}=\sqrt{{\left(\delta_{d,NS}-\delta_{d,SM}\right)}^{2}+{\left(\delta_{p,NS}-\delta_{p,SM}\right)}^{2}{\left(\delta_{h,NS}-\delta_{h,SM}\right)}^{2}}$$

From the values in Table 3, at higher IPA co-solvent content the computed term ∆*δ_NS−SM_* progressively decreases (from 9.29 MPa^1/2^ for pure DMF, to 6.13 MPa^1/2^ for a DMF/IPA solvent mixture of 70:30; from 11.3 MPa^1/2^ for pure NMP, to 7.24 MPa^1/2^ for a NMP/IPA solvent mixture of 70:30); therefore, the smaller difference in solubility parameters (i.e., higher affinity) between non-solvent and solvent mixtures results in a faster exchange between the non-solvent and the binary DMF/IPA or NMP/IPA solvent mixtures.

Membranes prepared using 100% DMF as a solvent-i.e., without co-solvent addition resulted in an approximately 2 min delay on demixing. The introduction of IPA into the dope solution decreased the delay and, when the IPA amount was increased to 30 wt%, instantaneous demixing was observed.

**Figure 8:**
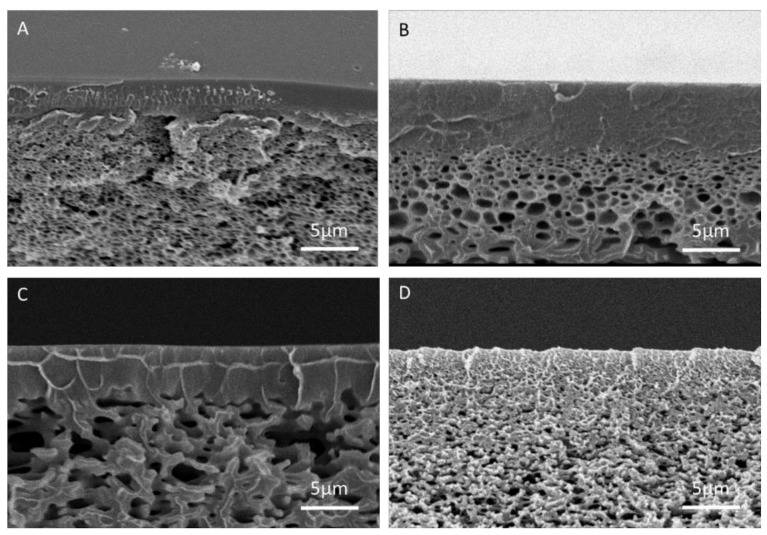
Cross section of membranes with solvent/co-solvent (DMF/IPA) ratios in the solvent mixture of (**A**) 100/0, (**B**) 90/10, (**C**) 80/20, (**D**) 70/30.

Cross-sectional micrographs of manufactured membranes are illustrated in Figure 8. Membranes exhibiting a dense top layer with a thickness of about 5 μm were formed when adding IPA to the dope solution at up to 20 *w*/*w*%. For higher IPA concentrations (20%), the dense top layer was replaced by a more open structure. In agreement with theoretical predictions, the porosity of the sub-layer increased at higher IPA contents in the solvent mixture: SEM images revealed a transition from a well-defined porous morphology to nodular and interconnected pores.

**Figure 9:**
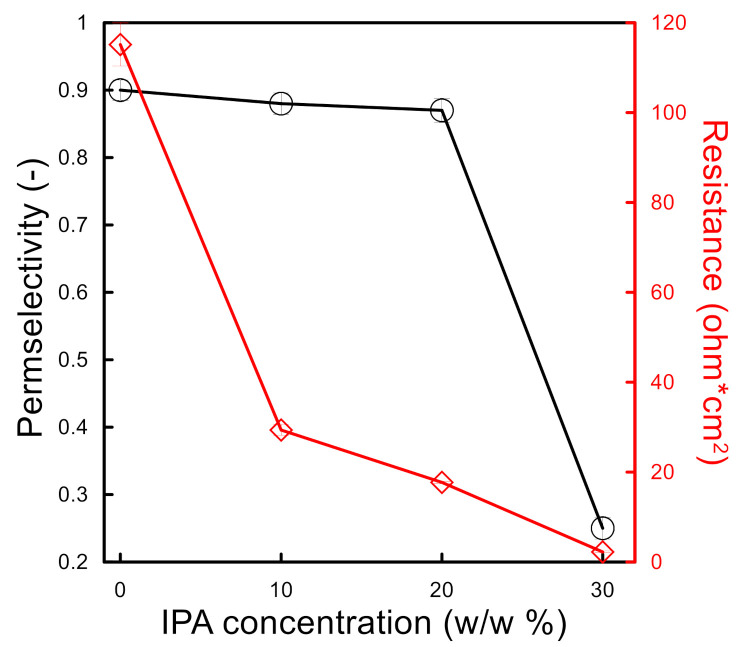
Electrochemical properties of SPSf/(DMF + IPA) membranes.

The permselectivity of the membranes prepared from SPSf/(DMF + IPA) solutions, measured for 0.1 M/0.5 M NaCl solution, is illustrated in Figure 9. Up to 20% IPA concentration in dope solution, the membranes exhibited an almost stable permselectivity close to 0.90; the latter drastically decreased down to 0.25 at 30% IPA. The findings from the electrochemical characterization are consistent with the morphological characteristics discussed above: the membranes with a dense top layer were able to exclude Cl^−^ ions, whereas those with a porous top layer allowed a massive transport of anions without any preferential selection.

Although the top layer thickness of the SPSf/(DMF + IPA) membranes, with IPA concentrations ≤ 20%, were not significantly different, the electrochemical resistance (measured for 0.5 M/0.5 M NaCl solutions) showed a noticeable decreasing trend for increasing IPA content (e.g., 75% reduction when adding 10% IPA to DMF solvent), coherently with a more open and interconnected porous structure of the sub-layer. As expected, the membrane prepared in the solvent DMF/IPA mixture 70:30 drastically lost its resistance due to the absence of the dense skin layer and interconnected pores throughout the cross-section.

**Figure 10:**
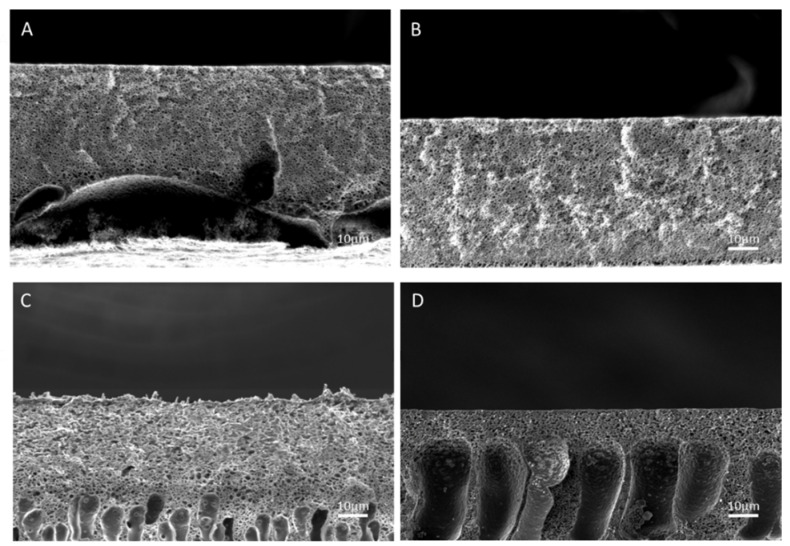
Cross section of membranes with solvent/co-solvent (NMP/IPA) ratios in the solvent mixture of (**A**) 100/0, (**B**) 90/10, (**C**) 80/20, (**D**) 70/30.

As shown in Figure 10, the membranes prepared using 100% NMP as a solvent exhibited an homogenous structure that was substantially nanoporous; if compared to SPSf membranes prepared from 100% DMF, this morphological behavior is well justified by the lower affinity of IPA for NMP with respect to DMF, which has a lower solvent/non-solvent exchange rate (see the discussion on Hansen Solubility Parameters). SEM cross-sectional images indicate that, as a consequence of the addition of IPA to the solvent mixture, the membrane morphology became asymmetric with the appearance of a fingerlike sublayer. Coherently, the dimension of fingerlike pores increased with an increasing concentration of IPA in the (NMP + IPA) solvent mixture (Figure 10B–D). In addition to that, the structure of the top layer clearly became nodular for an IPA content of 30% in the dope solution.

**Figure 11:**
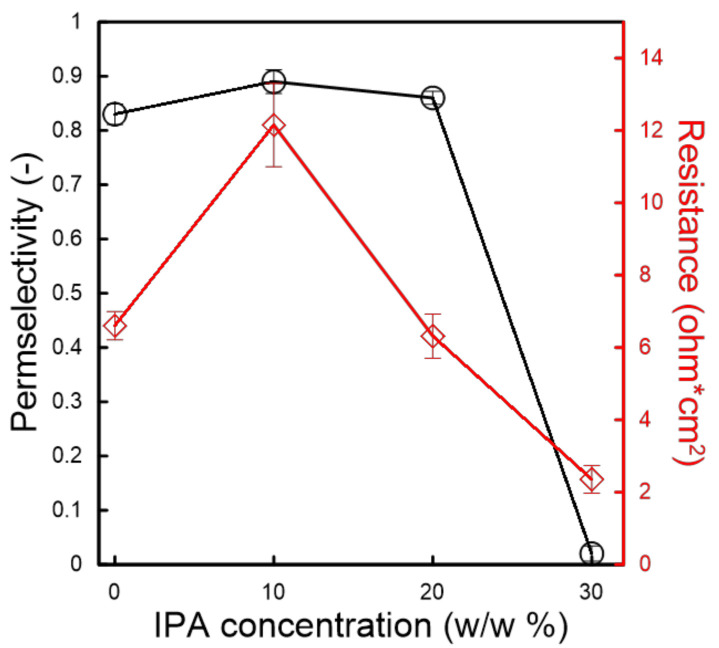
Electrochemical properties of SPSf/(NMP + IPA) membranes.

The measured pemselectivity of SPSf/(NMP + IPA) membranes, measured for 0.1 M/0.5 M NaCl solutions, is reported in Figure 11. Interestingly, despite the prevalent nanoporous structures of membranes with an IPA content in the solvent mixture ≤ 20%, the permselectivity remained almost stable, with values above 80%. The absence of interconnected pores through the cross section of CEMs is a likely explanation for this relatively high permselectivity for an open structure. A further increase in the IPA concentration up to 30% *w/w* resulted in a reduced permselectivity due to the formation of nodular pores, as the large interstitial channels are not able to exclude the co-ions.

The resistance of the membranes was found to be between 12.2 and 2.4 Ω∙cm^2^, with a decreasing trend at higher IPA contents in the solvent mixture except for the membrane prepared with pure NMP. Analyzing the electrochemical characterization and morphology together and comparing them with the properties of membranes prepared with DMF, it can be assumed that the membrane prepared with pure NMP is an outsider. Considering the properties of the remaining membranes, it can be inferred that the ionic resistance is directly related to the membrane morphology: the presence of large cavities and interconnected pores enhances the transport of the ions, so the resistance decreases (−81% when shifting from pure 10% *w*/*w* to a 30% *w*/*w* addition of IPA).

A convenient membrane for the RED application must have a high permselectivity and low electrical resistance. Current commercially available ion exchange membranes have a permselectivity >0.85 and a resistance from 0.4 to 30 Ω∙cm^2^ [36]. Considering the resistance range of commercial CEMs, the DMF-based membranes prepared in this study had an unacceptably high resistance despite their comparable permselectivity. On the other hand, the NMP-based membranes showed a promising performance; in particular, the membrane prepared with an NMP:IPA 80:20 solvent mixture, and hereinafter referred to as “SPSf” to simplify the notation, exhibited the best electrochemical properties. As a next step, the SPSf and the commercial benchmark Neosepta CMX were further analyzed under exposure to different concentrations of NaCl solution pairs—i.e., 0.1 M/0.5 M, 0.1 M/1.0 M, and 0.5 M/1.0 M (where 0.1 M, 0.5 M and 1.0 M NaCl solutions artificially replicate brackish water, seawater, and reverse osmosis brine salinity, respectively)—of interest for Reverse Electrodialysis (RED) applications.

**Figure 12:**
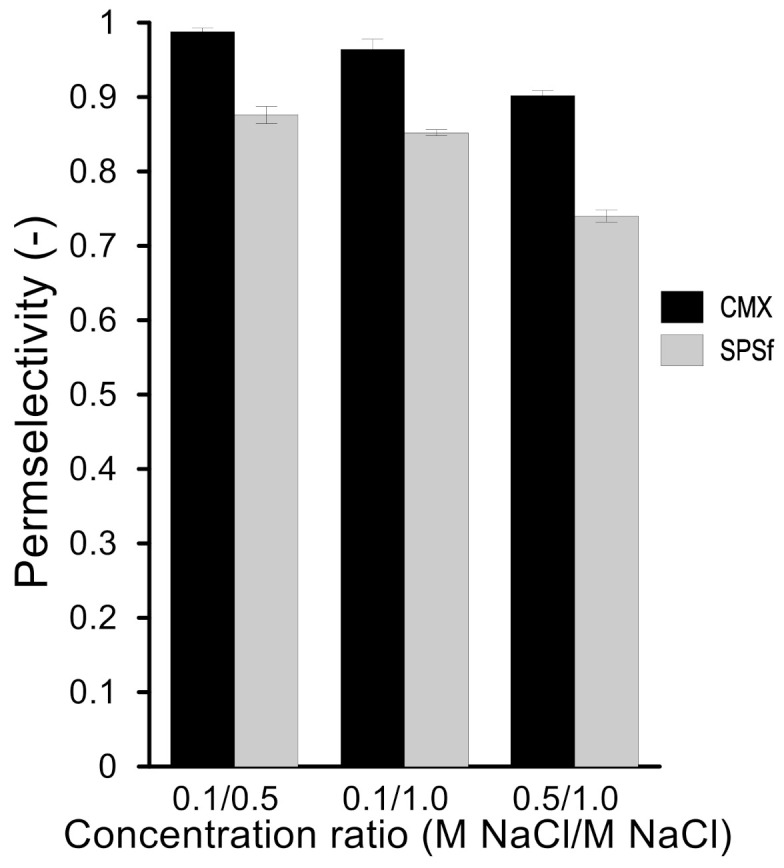
Permselectivity of CMX and SPSf exposed to different NaCl solution pairs.

The permselectivity (α) of CMX and SPSf responded in a similar fashion to the increasing solution concentration (Figure 12); in general, having more ions in the solutions reduced the co-ion exclusion capacity of the membranes (α_0.1/0.5_ > α_0.1/1.0_ > α_0.5/1.0_). The experimental results are consistent with the literature [37,38,39]. Increasing ion concentration caused a decreasing permselectivity due to inefficient Donnan exclusion. Furthermore, increasing the concentration gradient would result in a higher permselectivity when the highest concentration is kept constant [37].

**Figure 13:**
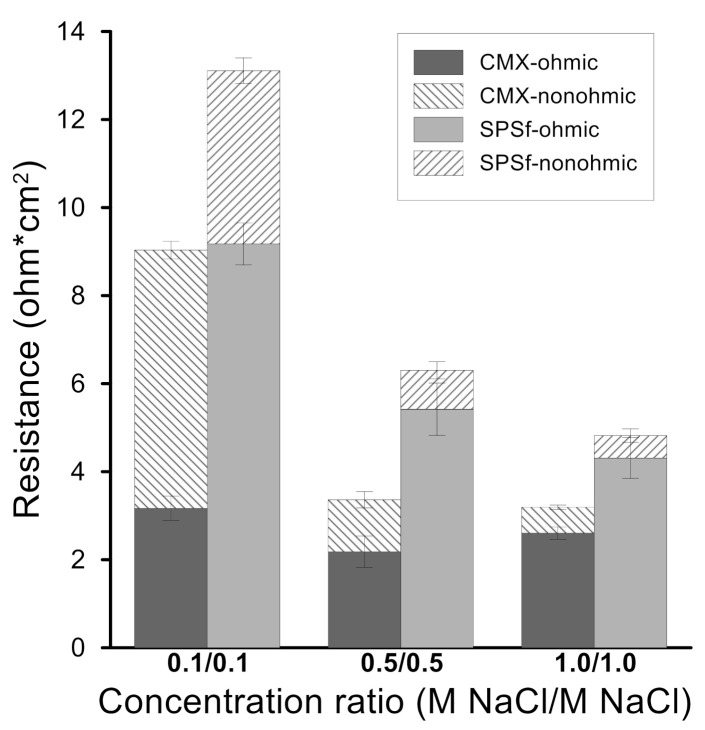
Resistance of CMX and sPSf at different NaCl concentrations.

The ohmic and non-ohmic resistances are presented in Figure 13 for increasing NaCl concentrations. For the SPSf membrane exposed to an increasing salt content, both the resistances progressively reduced, while, for the CMX membrane investigated under the same conditions, the ohmic resistance remained constant, whereas the non-ohmic resistance decreased. The different trend on ohmic resistance of two membranes is likely related to the membrane morphology: while CMX has a dense structure, the SPSf membranes exhibit an asymmetric structure with non-interconnected nanopores. Therefore, the resistance of SPSf is dominated by the salt solution present within the channel: the electrical resistivity of the salt solution decreases at higher NaCl concentrations and, ultimately, the ohmic resistance of the membrane falls. On the other hand, dense CMX membranes offer a limited intrusion of solution within their structure, and the ohmic resistance is largely determined by the polymeric barrier. The ohmic and non-ohmic resistances of SPSf resulted in 53% and 87% decreases, respectively, when the test solution concentration increased from 0.1 to 1.0 M NaCl. The ohmic and non-ohmic resistances obtained in this study are comparable to those of previous studies on the resistance of CMX membranes [40,41]. For example, Galama et al. (2014) studied the resistance of CMX in different solution concentrations ranging from 0.01 to 1.1 M NaCl; similar to our work, the ohmic resistances of CMX in 0.1, 0.5, and 1.1 M NaCl were 5.74, 3.13, and 3.41 Ω·cm^2^, respectively, while the non-ohmic resistances were 5.91, 0.30, and 0.37 Ω·cm^2^, respectively [41].

Although the SPSf membrane has a lower permselectivity and a higher resistance compared to the benchmark CMX membrane, the estimation of the RED power potential is worthwhile in the consideration of the potentially higher manufacturing simplicity and lower membrane cost of the novel SPSf membrane developed in this study.

The theoretical maximum power density was calculated as follows [42]:

(7) Pmax=αRTFlnacad22RCEM+dkc+dkd,

where α is permselectivity (−), *a_c_* is the ionic activity of the concentrated solution (−), *a_d_* is the ionic activity of the diluted solution (−), *R_CEM_* is the ionic resistance of CEM (Ω·m^2^), *d* is the thickness (m), *k_c_* is the conductivity of the concentrated solution (Ω·m), *k_d_* is the conductivity of the diluted solution (Ω·m), *F* is the Faraday constant (96500 s·A·mol^−1^), *T* is the temperature (298 K), and *R* is the universal gas constant (8.314 J·mol^−1^K^−1^). The theoretical calculation was made for a half-cell including half of the diluted compartment (250 µm), half of the concentrated compartment (250 µm), and the CEM.

**Figure 14:**
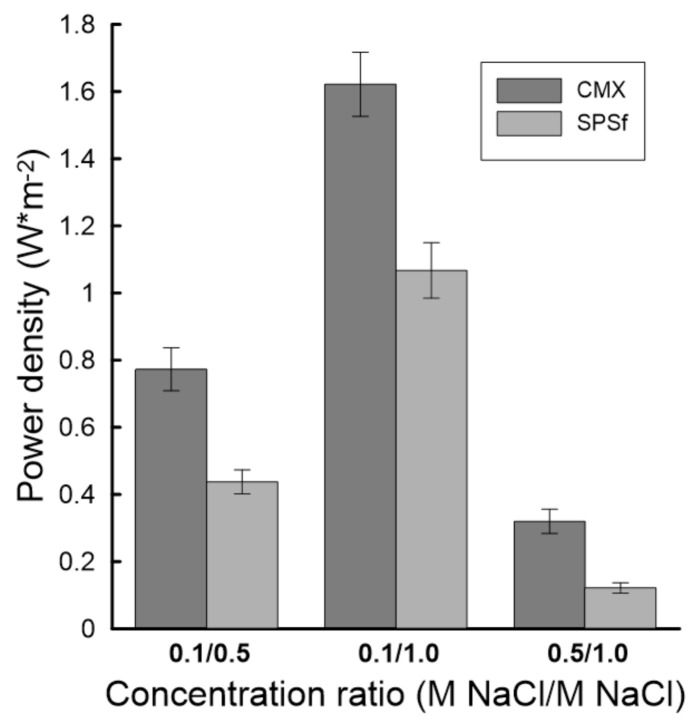
Theoretical power density of the CMX and sPSf membranes exposed to different NaCl solution pairs.

A summary of the power density calculation is presented in Figure 14 for the 0.1 M/0.5 M, 0.1 M/1.0 M, and 0.5 M/1.0 M NaCl solution pairs. The order of theoretical power density was in the same order of concentration gradient for both membranes—i.e., P_0.1/1.0_ > P_0.1/0.5_ > P_0.5/1.0_. The highest theoretical power density for SPSf was 1.08 W·m^−2^ for 0.1 M/1.0 M NaCl, corresponding to 66% of the value computed for CMX at the same condition. The performance of the SPSf membrane deteriorated at a lower salinity gradient, resulting in about 57% of the maximum power density generated by the CMX membrane for the 0.1 M/0.5 M NaCl solution pairs.

## 4. Conclusions and Outlook

In this study, PSf was successfully sulfonated to prepare novel asymmetric integral cation exchange membranes for application in Reverse Electrodialysis (RED). As a result of a parametric study carried out at different sulfonation degree, sulfonated polysulfone with 0.31 DS was selected considering the mechanical stability of membranes prepared via wet phase inversion. Aiming at the optimization of the electrochemical properties of cation exchange SPSf membranes as a function of their morphology, the experimental work was complemented by the preparation and characterization of membranes using two different main solvents (NMP, DMF) and a co-solvent (IPA) with the aim of appropriately modulating the exchange rate between the non-solvent (IPA) and the solvent mixture.

The most promising membrane—i.e., the one resulting from the SPSf dissolved in an 80:20 NMP/IPA solvent mixture was further characterized for different NaCl solution pairs (mimicking brackish water, seawater, and brine) to estimate the theoretical power generation potential under the RED operation. The SPSf membrane had a comparable permselectivity but a relatively high electrical resistance; as a result, the power density generated by the SPSf was lower than the one computed for the commercial Neosepta CMX cation exchange membrane assumed as a benchmark.

Despite their lower performance (although there is still room for further improvement by optimizing the wide set of phase separation parameters), SPSf membranes can be promising CEMs candidates in view of the potential commercialization of RED when one considers that membrane cost represents the most relevant barrier to market penetration. Preliminary studies indicate that a feasible RED operation requires a membrane cost lower than 5 €·m^−2^ [14,43]. In the present work, the methodological approach to the lab-scale preparation of membranes includes the cheap sulfonation procedure of an engineering polymer (price of CSA and PSf are 0.56 $/kg [44] and 2–7.5 $/kg [45]), low polymer consumption, easy scalable, and industrially assessed phase inversion process; this approach has the potential to bring the price of CEM membranes near to the identified target. Moreover, the electrochemical properties can be further enhanced by the investigation of other phase inversion parameters and diminishing the thickness of the selective layer.

## Figures and Tables

**Table 1:** Activity coefficient and theoretical membrane potential of the test solution pairs [27].

Concentration (M NaCl)	Activity Coefficient (−)	NaCl Solutions Pair (M/M)	Δ*V_theoretical_* (mV)
0.1	0.778	0.1/0.5	37.9
0.5	0.681	0.1/1.0	54.8
1.0	0.657	0.5/1.0	16.9

**Table 2:** Hansen Solubility Parameters of DCM, PSf, and SPSf [31,32].

Component	δ_d_ (MPa^1/2^)	δ_p_ (MPa^1/2^)	δ_h_ (MPa^1/2^)
DCM	18.2	6.3	6.1
PSf (DS = 0)	18.2	4.3	6.9
SPSf (DS = 0.30)	18.2	5.0	8.5
SPSf (DS = 1.00)	18.1	6.0	11.0

**Table 3:** Hansen Solubility Parameters of pure solvents and their binary mixtures with co-solvent investigated in this study [32].

Solvent	δ_d_ (MPa^1/2^)	δ_p_ (MPa^1/2^)	δ_h_ (MPa^1/2^)
DMF	17.4	13.7	11.3
NMP	18.0	12.3	7.2
**Co-solvent**	**δ_d_ (MPa^1/2^)**	**δ_p_ (MPa^1/2^)**	**δ_h_ (MPa^1/2^)**
IPA	15.8	6.1	16.4
**Solvent Mixture**	**δ_d_ (MPa^1/2^)**	**δ_p_ (MPa^1/2^)**	**δ_h_ (MPa^1/2^)**
DMF/IPA (90/10)	17.2	12.8	11.9
DMF/IPA (80/20)	17.0	11.9	12.5
DMF/IPA (70/30)	16.9	11.1	13.0
NMP/IPA (90/10)	17.7	11.5	8.37
NMP/IPA (80/20)	17.4	10.8	9.47
NMP/IPA (70/30)	17.2	10.1	10.5

## Data Availability

Data is contained within the article.

## Abbreviations

AC	Alternating current
AEM	Anion exchange membranes
CEM	Cation exchange membranes
CSA	Chlorosulfonic acid
DC	Direct current
DCM	Dichloromethane
DMF	Dimethyl formamide
DMSO	Dimethyl sulfoxide
DS	Degree of sulfonation
IEC	Ion exchange capacity
IEM	Ion exchange membranes
IPA	2-propanol
NMP	1-Methyl-2-pyrrolidone
NS	Non-solvent
P	Polymer
PSf	Polysulfone
RED	Reverse electrodialysis
RAC	Alternative current resistance
RBL	Boundary layer resistance
RDC	Direct current resistance
RM	Membrane resistance
RS	Solution resistance
S	Solvent
SD	Swelling degree
SM	Solvent mixture
SPSf	Sulfonated polysulfone
